# The Effectiveness of Wearable Devices as Physical Activity Interventions for Preventing and Treating Obesity in Children and Adolescents: Systematic Review and Meta-analysis

**DOI:** 10.2196/32435

**Published:** 2022-04-08

**Authors:** Wentao Wang, Jing Cheng, Weijun Song, Yi Shen

**Affiliations:** 1 Department of Basic Education Zhejiang Tongji Vocational College of Science and Technology Hangzhou China

**Keywords:** wearable devices, obesity, children, adolescents, meta-analysis

## Abstract

**Background:**

The prevalence of obesity in children and adolescents remains a global public health issue. Wearable devices may offer new opportunities for prevention and intervention in obesity. Previous systematic reviews have only examined the effect of the wearable device interventions on preventing and treating obesity in adults. However, no systematic review has provided an evaluation of wearable devices as physical activity interventions for preventing and treating obesity in children and adolescents.

**Objective:**

The purpose of this review and meta-analysis was to evaluate the effectiveness of wearable devices as physical activity interventions on obesity-related anthropometric outcomes in children and adolescents.

**Methods:**

Research articles retrieved from PubMed, EMBASE, Cochrane Library, Scopus, and EBSCO from inception to February 1, 2021, were reviewed. The search was designed to identify studies utilizing wearable devices for preventing and treating obesity in children and adolescents. The included studies were evaluated for risk of bias following the Cochrane recommendation. Meta-analyses were conducted to evaluate the effectiveness of wearable devices as physical activity interventions on body weight, body fat, BMI z-score (BMI-Z), BMI, and waist circumference. Subgroup analyses were performed to determine whether the characteristics of the interventions had an impact on the effect size.

**Results:**

A total of 12 randomized controlled trials (3227 participants) were selected for meta-analysis. Compared with the control group, wearable device interventions had statistically significant beneficial effects on BMI (mean difference [MD] –0.23; 95% CI –0.43 to –0.03; *P*=.03; *I^2^*=2%), BMI-Z (MD –0.07; 95% CI –0.13 to –0.01; *P*=.01; *I^2^*=81%), body weight (MD –1.08; 95% CI –2.16 to –0.00; *P*=.05; *I^2^*=58%), and body fat (MD –0.72; 95% CI –1.19 to –0.25; *P*=.003; *I^2^*=5%). However, no statistically significant effect was found on waist circumference (MD 0.55; 95% CI –0.21 to 1.32; *P*=.16; *I^2^*=0%). The subgroup analysis showed that for participants with overweight or obesity (MD –0.75; 95% CI –1.18 to –0.31; *P*<.01; *I^2^*=0%), in the short-term (MD –0.62; 95% CI –1.03 to –0.21; *P*<.01; *I^2^*=0%), wearable-based interventions (MD –0.56; 95% CI –0.95 to –0.18; *P*<.01; *I^2^*=0%) generally resulted in greater intervention effect size on BMI.

**Conclusions:**

Evidence from this meta-analysis shows that wearable devices as physical activity interventions may be useful for preventing and treating obesity in children and adolescents. Future research is needed to identify the most effective physical activity indicators of wearable devices to prevent and treat obesity in children and adolescents.

## Introduction

With the development of society and technology, human lifestyles have undergone tremendous changes. The worldwide prevalence of obesity has risen rapidly since 1975. In 2016, more than 340 million children and adolescents aged 5-19 were overweight or obese [1]. As a global public health issue, obesity might cause a number of serious health conditions, such as high blood pressure, nonalcoholic fatty liver disease, abnormal lipid metabolism, and psychosocial problems [2-5]. Therefore, effective interventions for preventing and treating obesity in children and adolescents are urgently needed.

At present, regular physical activity seems to be one of the effective means for the prevention of and intervention in obesity among children and adolescents and has been discussed in many studies [6-8]. Traditional physical activity intervention methods (such as school group physical activity interventions [9] and face-to-face interventions with health professionals [10]) require significant effort and costs. However, wearable devices may provide an alternative means of addressing obesity in children and adolescents. Wearable devices, such as pedometers, sports bracelets, sports watches, and accelerometers, can offer easy and effective ways to collect physical activity data (steps, heart rate, energy expenditure, physical activity, and physical activity time of different intensity) and allow users to monitor their data [11,12]. These quantitative data can stimulate the user’s motivation for physical activity, increase physical activity time, and increase energy expenditure, thus enabling weight loss [13-18].

A considerable body of work related to the use of wearable devices to prevent and treat obesity has been already published [19-23]. Most previous reviews have focused on adults, in which it was demonstrated that wearable devices can achieve a significant effect size in reducing the BMI of adults with obesity (*β*=−1.57; *P*<.001) or adults with chronic diseases (*β*=−1.30; *P*<.001). However, evidence on the effectiveness of wearable device interventions remains inconclusive. Furthermore, wearable devices have no significant effect size on adults with normal weight (*β*=−0.49; *P*=.07) [17,24-26]. The characteristics and cognitive abilities of children and adolescents are different from those of adults, and emerging technologies have great appeal to children and adolescents [27,28]. Children and adolescents are in a sensitive period of growth and development. Compared with diet control, children and adolescents are more suited to perform increased physical activity for preventing and treating obesity [29,30]. Wearable devices have been proven to be valid and accurate [31-35], providing consistent feedback and inducing behavior changes in individuals, which result in increased physical activity [36-41]. Therefore, there is a need to explore the effectiveness of wearable devices as physical activity interventions for preventing and treating obesity in this population.

To the best of our knowledge, there are no reviews evaluating wearable devices as physical activity interventions for preventing and treating obesity in children and adolescents. It is thus necessary to explore the effectiveness of wearable devices as physical activity interventions to prevent and treat obesity specifically in these populations. The results may contribute to public health guidance on the use of wearable devices for addressing obesity in children and adolescents. Therefore, the objectives of this review and meta-analysis are to (1) evaluate the effectiveness of wearable devices as physical activity interventions on obesity-related anthropometric outcomes in children and adolescents and (2) determine whether the characteristics of the interventions had an impact on the effect size through subgroup analyses.

## Methods

### Study Design

This systematic review and meta-analysis is reported in accordance with the Preferred Reporting Items for Systematic Review and Meta-Analyses (PRISMA) guidelines (Multimedia Appendix 1) [42].

### Search Strategy

The following 5 international electronic databases were searched to discover studies on the use of wearable devices for preventing and treating obesity in children and adolescents: PubMed, EMBASE, Cochrane Library, Scopus, and EBSCO. The time span was set from the inception of each database to February 1, 2021. Search strategies were adapted according to the requirements of each database (see Multimedia Appendix 2 for the complete PubMed search strategy). At the same time, relevant articles were also found by checking the references of the included articles and previous systematic reviews.

### Inclusion and Exclusion Criteria

The inclusion criteria included studies with the following characteristics:

1. Population: The participants were children and adolescents aged 6-18 years.
2. Interventions: The intervention groups involved the use of wearable devices to promote physical activity. Wearable devices need to be worn on the user’s body, and use accelerometers or sensors to track the wearer’s physical activity or physiological data, such as wristbands, pedometers, smartwatches.
3. Outcomes: The outcome featured obesity-related anthropometric indicators, such as BMI, BMI z-score (BMI-Z), body weight, or body fat.
4. The experimental design was a randomized controlled trial (RCT).

The exclusion criteria were as follows:

1. The participants were aged <6 or >18 years.
2. The intervention did not involve wearable devices (eg, smartphones, video games, and social media) or their use was not related to promoting physical activity (eg, monitor food consumption and strengthen communication and guidance).
3. The primary outcomes were not obesity-related anthropometric indicators (eg, quality of life, food consumption, and psychological state).
4. The experimental design was not an RCT.
5. The articles were meta-analyses or systematic reviews.

The literature screening was first conducted independently by 2 authors (WS and YS), according to the inclusion and exclusion criteria described above. Then the 2 researchers cross-checked the included literature. Following this, documents for which eligibility was unclear were selected according to consensus of a third author (WW).

### Data Extraction and Risk of Bias Assessment

Two reviewers (WS and YS) extracted data independently from each included study. The extracted content included author, region, year of publication, clinical research design, research object, sample size, population characteristics, intervention method, intervention period, and outcome indicators.

Two reviewers (JC and YS) independently evaluated each study for risk of bias following the Cochrane recommendations. Each criterion was scored as having a low, unclear, or high level of risk. The evaluation content included (1) allocation concealment, (2) random sequence generation, (3) blinding of the outcome, (4) blinding of participants and personnel, (5) selective reporting, (6) incomplete outcome data, and (7) other bias [43].

### Statistical Analysis

RevMan5.3 software (International Cochrane Collaboration) was used for the meta-analysis. The mean difference (MD) and 95% CI were used to represent continuous variables. First, the included studies were tested for heterogeneity at a level of OR of 0.05. When *I^2^*≤50%, there is no statistically significant heterogeneity and the fixed effects model should be used; when *I^2^*>50%, there is a statistical heterogeneity between the studies and the random effects model should be used. For studies with heterogeneity, a sensitivity or subgroup analysis could be carried out; for studies with clinical heterogeneity and methodological heterogeneity, a meta-analysis of outcome indicators was abandoned, and a general statistical description was used.

## Results

### Study Selection

**Figure 1:**
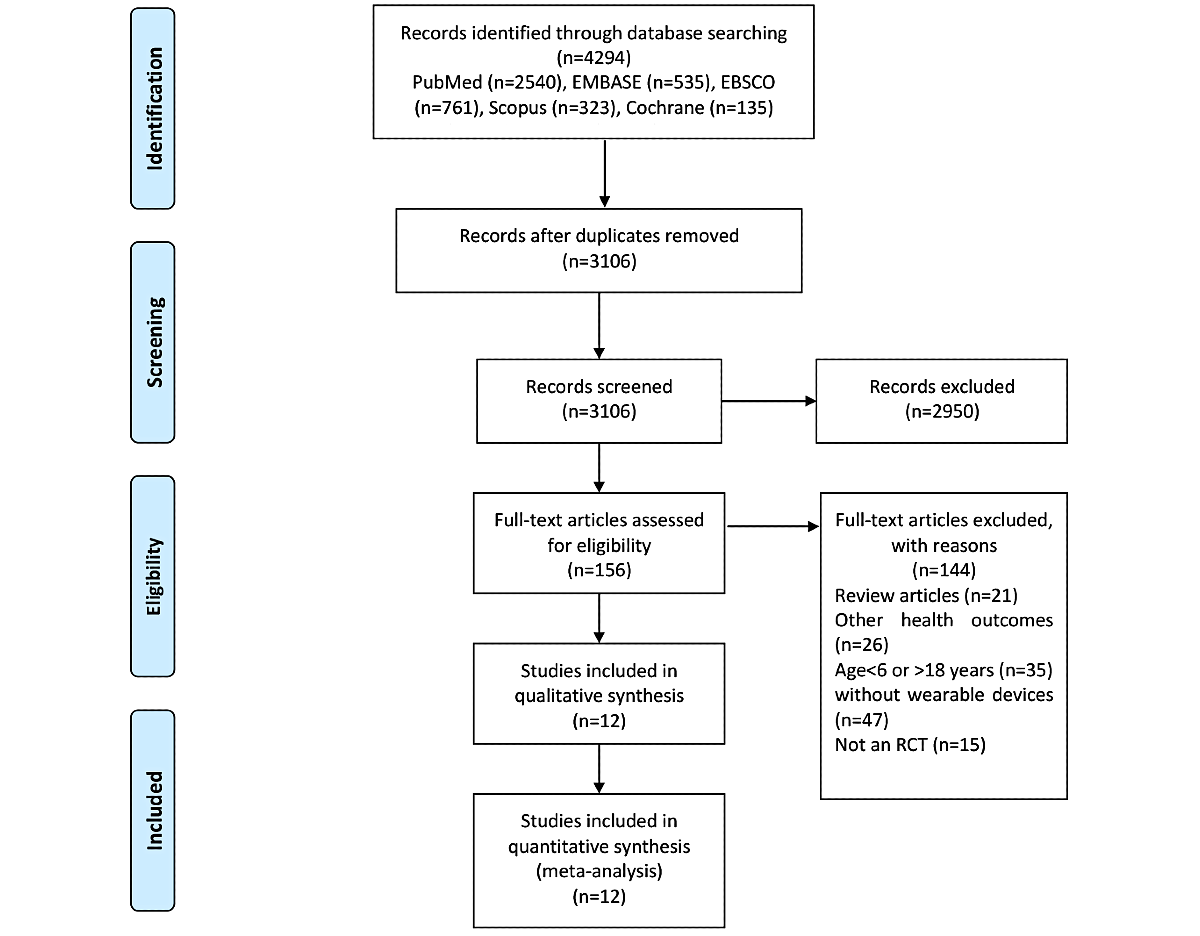
The selection process for the systematic review and meta-analysis. RCT: randomized controlled trial.

The flowchart in Figure 1 outlines the process for selecting articles for inclusion. A total of 4294 related articles were identified from 5 databases. A total of 1188 articles were eliminated as duplicates, and a further 2950 records were eliminated after screening of titles and abstracts. The remaining 156 articles were reviewed in detail and evaluated based on their full text. A further 26 articles were eliminated because the outcomes did not include obesity indicators, 35 owing to their irrelevance to children or adolescents, 47 for not featuring wearable devices, and 15 because their experimental design was not an RCT. Finally, 12 articles were selected for this meta-analysis.

### Risk-of-Bias Assessment

The risk-of-bias assessment results are presented in Multimedia Appendix 3. All studies were assessed as having a low risk of bias in terms of blinding of the outcome and selective reporting. In terms of random sequence generation, 11 studies had a low risk of bias, and only 1 study had a high risk of bias [44]. In the allocation concealment, 10 studies had a high risk of bias or were unclear, and 2 studies had a low risk of bias [45,46]. In terms of blinding of participants and personnel, all studies had a high risk of bias, due to the nature of the intervention. In the incomplete outcome data, 4 studies had a high risk of bias [46-49], all of which had a high rate of attrition (>25%). As many as 2 studies had a high risk of other bias [47,49], because the baseline of the intervention group and the control group was significantly different (*P*=.02).

### Characteristics of Included Studies

The characteristics of the included studies are presented in Multimedia Appendix 4. A many as 4 studies were carried out in the United States [48-51], 4 in Australia [44,46,52,53], 1 in Finland [23], 1 in Italy [47], 1 in Germany [54], and 1 in Singapore [45]. These included studies were published between 2011 and 2021 and involved 3227 participants. The average age of participants was 13.2 years and ranged from 6 to 18 years. The dropout rate was studied for each intervention and ranged from 3.9% (4/102) to 52.0% (25/48), with the average being 19.27% (622/3227). Intervention duration ranged from 2.5 to 18 months, with the average being 6.2 months. Among these 12 studies, 8 intervention targets were people of normal weight [23,44-46,50,52-54] and 4 were those with overweight or obesity [47-49,51]. A total of 7 studies used pedometers [44-46,49,52-54] and the other 5 used wristband activity trackers [23,47,48,50,51]. The most common outcome index was BMI [23,44-46,48-53], followed by BMI-Z [46,47,49,51-53], body fat [23,44,50,52-54], body weight [23,47-49,52], and waist circumference [23,44,46,48,52]. There were 2 wearable device intervention groups in the same studies [49,50]. The data from each intervention group were included in the meta-analysis as independent samples.

### Effects of Intervention

#### BMI

**Figure 2:**
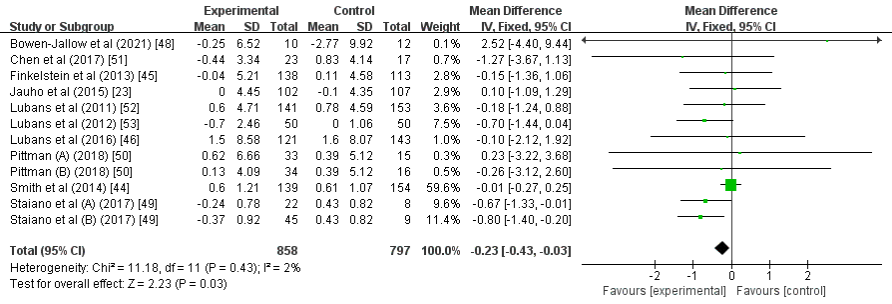
Forest plot of the effect of the wearable device interventions on BMI.

A total of 10 studies explored the effects of wearable devices as physical activity interventions on the BMI of children and adolescents [23,44-46,48-53]. There were statistically significant decreases in BMI between the group with wearable device interventions and the control group (MD –0.23; 95% CI –0.43 to –0.03; *P*=.03); heterogeneity was low and insignificant (*I^2^*=2%; *P*=.43; Figure 2). In this analysis, the study conducted by Smith et al [44] had the greatest proportion (59.6%).

**Figure 3:**
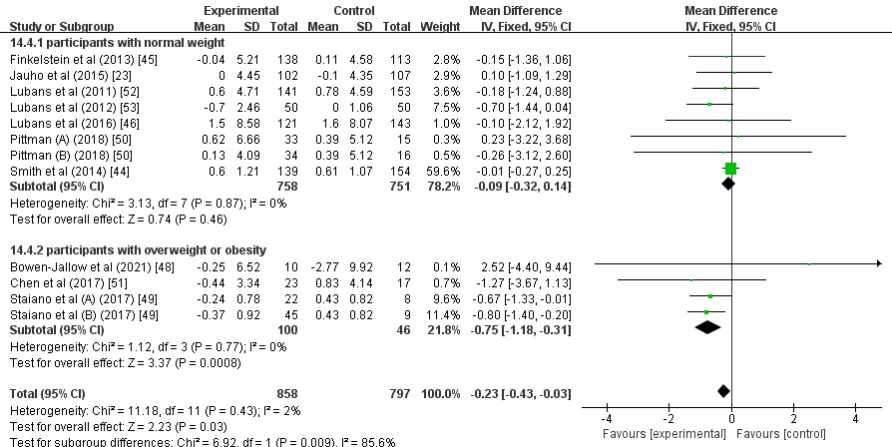
Forest plot of the participant characteristics subgroup analysis.

**Figure 4:**
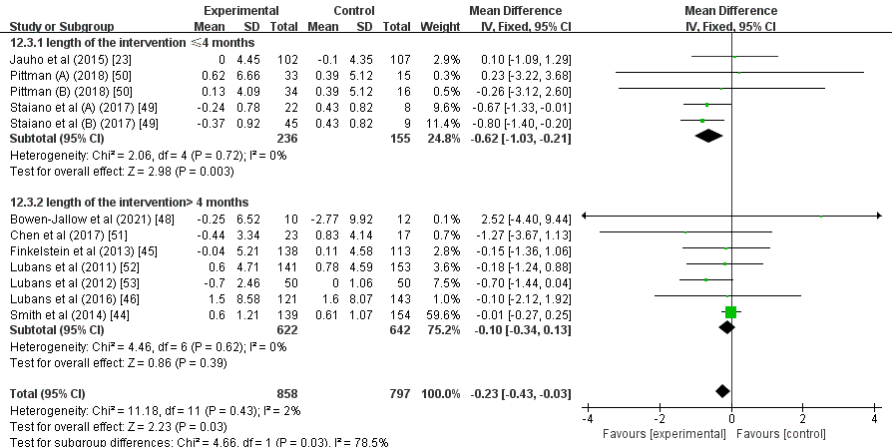
Forest plot of the intervention duration subgroup analysis.

**Figure 5:**
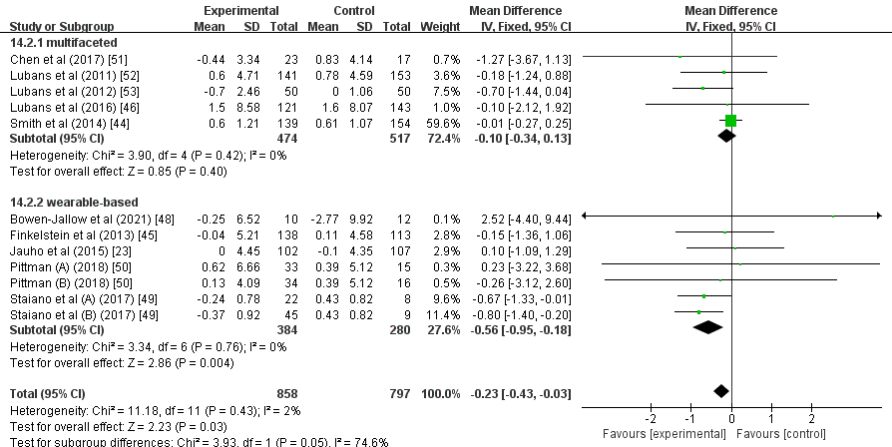
Forest plot of the intervention program subgroup analysis.

A subgroup analysis found that, compared with participants with normal weight (MD –0.09; 95% CI –0.32 to 0.14; *P*=.46; *I^2^*=0%; Figure 3), participants who were overweight or obese had a significantly greater intervention effect size on BMI (MD –0.75; 95% CI –1.18 to –0.31; *P*<.01; *I^2^*=0%). Another subgroup analysis showed that interventions with an estimated duration of ≤4 months (MD –0.62; 95% CI –1.03 to –0.21; *P*<.01; *I^2^*=0%; Figure 4) had a significantly greater intervention effect size on BMI than those with an estimated duration of >4 months (MD –0.10; 95% CI –0.34 to 0.13; *P*=.39; *I^2^*=0%). Further subgroup analysis demonstrated that, compared with the multifaceted intervention program (MD –0.10; 95% CI –0.34 to 0.13; *P*=.40; *I^2^*=0%; Figure 5), the wearable-based intervention programs caused a significantly greater decrease in BMI (MD –0.56; 95% CI –0.95 to –0.18; *P*<.01; *I^2^*=0%).

#### BMI-Z

**Figure 6:**
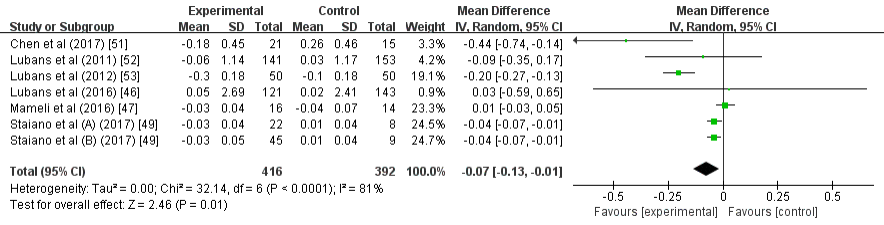
Forest plot of the effect of the wearable device interventions on BMI-Z (BMI z-score).

A total of 6 studies explored the effects of wearable devices as physical activity interventions on the BMI-Z of children and adolescents [46,47,49,51-53]. There were statistically significant decreases in BMI-Z between the groups with wearable device interventions and the control group (MD –0.07; 95% CI –0.13 to –0.01; *P*=.01); heterogeneity was high and significant (*I^2^*=81%; *P*<.01; Figure 6). Further sensitivity analysis was carried out. When the study of Lubans et al [53] was excluded, the effects of the intervention study on the BMI-Z showed a significant change (MD –0.03; 95% CI –0.07 to 0.01; *P*=.10; *I^2^*=57%). Here, the Staiano et al [49] study had the greatest proportion (49.2%).

#### Body Weight

**Figure 7:**
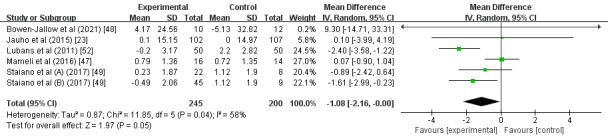
Forest plot of the effect of the wearable device interventions on body weight.

A total of 5 studies explored the effects of wearable devices as physical activity interventions on the body weight of children and adolescents [23,47-49,52]. Statistically significant decreases in body weight between the group with wearable device interventions and the control group were found (MD –1.08; 95% CI –2.16 to 0.00; *P*=.05); heterogeneity was high and significant (*I^2^*=58%; *P*=.04; Figure 7). According to the results of further sensitivity analysis, after excluding the study performed by Mameli et al [47], the heterogeneity of the intervention study showed a significant change (MD –1.69; 95% CI –2.45 to –0.93; *P*<.01; *I^2^*=0%). Among the studies, that by the Mameli et al [47] had the greatest proportion (27.0%).

#### Waist Circumference

**Figure 8:**
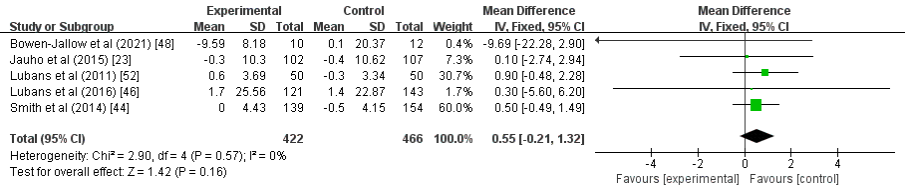
Forest plot of the effect of the wearable device interventions on waist circumference.

A total of 5 studies explored the effects of wearable devices as physical activity interventions on the waist circumference of children and adolescents [23,44,46,48,52]. Compared with the control group, the groups with wearable device interventions did not present statistically significant decreases in waist circumference (MD 0.55; 95% CI –0.21 to 1.32; *P*=.16); heterogeneity was low and insignificant (*I^2^*=0%; *P*=.57; Figure 8). The study by Smith et al [44] had the greatest proportion in this analysis (60.0%).

#### Body Fat Percentage

**Figure 9:**
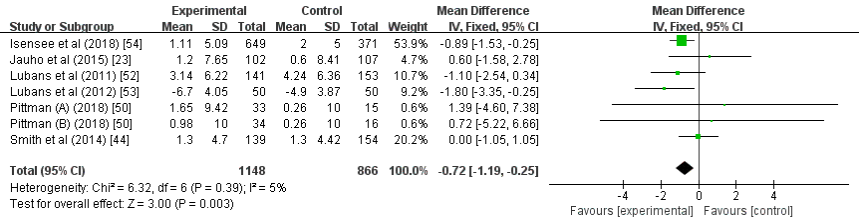
Forest plot of the effect of the wearable device interventions on body fat percentage.

A total of 6 studies examined the effects of wearable devices as physical activity interventions on the body fat of children and adolescents [23,44,50,52-54]. There were statistically significant decreases in body fat between the group with wearable device interventions and the control group (MD –0.72; 95% CI –1.19 to –0.25; *P*=.003); heterogeneity was low and insignificant (*I^2^*=5%; *P*=.39; Figure 9). The study by Isensee et al [54] had the greatest proportion (53.9%).

## Discussion

### Principal Findings

This review and meta-analysis synthesized the existing evidence on the effectiveness of wearable devices as physical activity interventions on obesity-related outcomes in children and adolescents. The results indicated that, compared with the control group, wearable device interventions have statistically significant effects on BMI, BMI-Z, body weight, and body fat. However, no statistically significant effects on waist circumference were found. The subgroup analysis showed that for participants with overweight or obesity, in the short term, wearable-based interventions had a significantly greater intervention effect size on BMI.

This review demonstrates that the use of wearable devices as physical activity interventions can statistically significantly improve the BMI, BMI-Z, body weight, and body fat of children and adolescents. This is consistent with the results of other systematic reviews. Two previous reviews that examined the effectiveness of wearable devices as physical activity interventions for adults found that after using wearable devices, there were statistically significant improvements in obesity-related outcomes such as BMI and body weight [17,25]. These conclusions further confirmed the effect of physical activity on preventing and treating obesity. These conclusions also demonstrate that physical activity plays an important role in the effectiveness of wearable devices for preventing and treating obesity. According to the behavior intervention technology model, wearable devices can promote physical activity through self-monitoring, goal setting, feedback, and motivation enhancement [55]. Assuming no drastic changes in dietary behavior, increasing physical activity, especially of moderate and vigorous intensity, will contribute to a negative caloric balance, leading to weight loss and BMI reduction [13,15,25]. Future research is needed to further explore what are the most effective physical activity indicators (eg, step count, total physical activity, and moderate-to-vigorous intensity physical activity) of wearable devices to prevent and treat obesity.

Four previous reviews examined the effectiveness of mobile health technology interventions in preventing and treating obesity in children and adolescents [56-59], finding that mobile health technology interventions yielded no significant improvements in obesity-related anthropometric outcomes such as BMI and body weight. These 4 reviews mixed different intervention strategies (promoting physical activity, monitoring food consumption, and strengthening communication and guidance) to explore the effects of mobile health technologies on prevention and treatment in obesity. By contrast, our review only concentrated on a single intervention strategy (promoting physical activity). These results may aid in the formulation of the most suitable intervention strategies on the use of mobile health technologies for addressing obesity among children and adolescents.

Waist circumference is a common indicator of abdominal obesity. This meta-analysis indicated that the wearable devices as physical activity interventions had no significant effect on waist circumference. This result is different to that of another meta-analysis in adults [17]. The reason may be that children and adolescents are in a sensitive period of growth and development, and waist circumference tends to increase, which will counteract part of the intervention effect [60]. It is worth noting that neither of these 2 systematic reviews included more than 5 RCTs. Thus, these conclusions must be treated cautiously.

A subgroup analysis found that, compared with participants with normal weight, those who were overweight or obese had a significantly greater intervention effect size on BMI. This result is consistent with other meta-analyses [17,25,26]. The reason may be that, compared with people with normal weight, the lifestyle of people with obesity is normally accompanied by more sedentary behaviors and less physical activity, and the level of physical activity is lower than that recommended by the World Health Organization [61,62]. Wearable devices can quantify the gap between the current activity level and the recommended amount [12,34]. Such a quantitative gap can motivate individuals to improve their physical activity level, and users can utilize self-regulation to change their physical activity habits as well as sedentary behaviors to manage their weight [13,63-67]. Wearable devices have achieved weight controlling effects by prompting a change in the lifestyle of people with obesity. For people of normal weight, the quantified physical activity level is not much different from the recommended amount. It cannot stimulate awareness of any deficiencies in their own activity, so the intervention effect is not obvious [68]. These facts suggest that the same intervention program may have different effects on different populations. Therefore, we need to analyze the baseline physical activity levels of different populations and formulate targeted intervention programs to achieve better intervention effects.

Another subgroup analysis found that interventions with a duration of ≤4 months had a significantly greater effect on BMI than those with a duration of >4 months. This is supported by the other systematic literature reviews [57,69], which showed that it is difficult for people to maintain focus on technology over time. Beyond 4 months, the freshness and interest in wearable devices disappear, resulting in the gradual disappearance of the intervention’s effect [70]. However, 2 meta-analyses previously reported that wearable device interventions with the duration exceeding 4 months can achieve better BMI reduction [17,66]. It was preliminarily evidenced that children and adolescents were prone to losing interest and had poor compliance [71]. When using wearable devices for long-term interventions, targeted strategies should be applied at different periods of the intervention (such as self-monitoring in the first 3 months, peer competition at 3-6 months, family incentives at 6-9 months, and cash rewards at 9-12 months) so that children and adolescents can effectively maintain long-term interest and compliance.

The subgroup analysis clarified that, compared with the multifaceted intervention program, the wearable-based intervention program had a significantly greater impact on BMI. Wearable-based interventions focused on improving the user’s physical activity levels to achieve weight loss. However, multifaceted interventions are based on multiple components. On the one hand, the intervention strategies did not focus on improving the physical activity level through wearable devices. On the other hand, the primary goal of intervention was not to prevent or treat obesity [37,57]. Therefore, the multifaceted intervention had a limited ability to reduce BMI. This suggests the need to focus on improving physical activity levels through wearable devices to prevent and treat obesity in children and adolescents.

### Strengths and Limitations

Our systematic review has some strengths. First, to the best of our knowledge, this study may be the first meta-analysis to summarize the evidence on the effects of wearable devices as physical activity interventions on preventing and treating obesity in children and adolescents. Second, we chose the intervention tool focused on wearable devices, which are the latest mobile health technology products with advantages in functionality and convenience. Third, our review concentrated on a single intervention strategy (promoting physical activity through wearable devices). Fourth, the included studies were all RCTs with high-level evidence. Finally, this systematic review performed subgroup analyses to determine whether the characteristics of the interventions had an impact on the effect size.

The limitations of our review results must be clarified. First, relatively few studies met our inclusion criteria. This made it difficult to draw any definite conclusions. Second, 4 studies were found to have a high risk of bias of incomplete outcome data, for which the dropout rates were >25%. Third, the high heterogeneity of the BMI-Z and body weight indicators in this meta-analysis cannot be ignored. Finally, in the meta-analysis of obesity-related anthropometric indicators, individual studies occupied excessive proportion in the analysis. Accordingly, the results of the meta-analysis may be affected by a single study.

### Conclusions

This meta-analysis indicated that the use of wearable devices as physical activity interventions can significantly reduce BMI, BMI-Z, body weight, and body fat in children and adolescents, but failed to significantly improve waist circumference. The subgroup analysis showed that for participants with overweight or obesity, in short term, wearable-based interventions generally resulted in greater improvements in BMI. Therefore, wearable devices as physical activity interventions may be useful for preventing and treating obesity in children and adolescents. Future research is needed to identify the most effective physical activity indicators of wearable devices to prevent and treat obesity in children and adolescents.

## Appendix

Multimedia Appendix 1 PRISMA (Preferred Reporting Items for Systematic Review and Meta-Analysis) checklist.

Multimedia Appendix 2 PubMed search strategy.

Multimedia Appendix 3 Risk of bias summary.

Multimedia Appendix 4 Characteristics of included studies.

## Abbreviations

PRISMA: Preferred Reporting Items for Systematic Review and Meta-Analysis
RCT: randomized controlled trial
