# Supplementary material for: The Effectiveness of Wearable Devices as Physical Activity Interventions for Preventing and Treating Obesity in Children and Adolescents: Systematic Review and Meta-analysis
Source: JMIR Mhealth Uhealth. 2022 Apr 8;10(4):e32435. doi: 10.2196/32435 (PMC9034426; doi:10.2196/32435)
Supplement: Multimedia Appendix 2 [file mhealth_v10i4e32435_app2.docx]

# Multimedia Appendix 2. PubMed search strategy

| **PubMed** | | |
| --- | --- | --- |
| #4 | Search: (((((((((((((((((((((((((Wearable Electronic Devices) OR (Device, Wearable Electronic)) OR (Devices, Wearable Electronic)) OR (Electronic Device, Wearable)) OR (Electronic Devices, Wearable)) OR (Wearable Electronic Device)) OR (Wearable Technology)) OR (Technologies, Wearable)) OR (Technology, Wearable)) OR (Wearable Technologies)) OR (Wearable Devices)) OR (Device, Wearable)) OR (Devices, Wearable)) OR (Wearable Device)) OR (Electronic Skin)) OR (Skin, Electronic)) OR (wristbands)) OR (smartwatches)) OR (Fitbit)) OR (Sports bracelet)) OR (physical activity tracker)) OR (accelerometer)) OR (electronic bracelet)) OR (Pedometer)) AND ((((((adolescent) OR (child)) OR (teen)) OR (youth)) OR (minors)) OR (Pediatric))) AND ((((((((((obesity) OR (obese)) OR (overweight)) OR (weight loss)) OR (BMI)) OR (body mass index)) OR (body weight)) OR (weight management)) OR (Body Composition)) OR (Waist circumference)) | 2,540 |
| #3 | Search: (((((((((obesity) OR (obese)) OR (overweight)) OR (weight loss)) OR (BMI)) OR (body mass index)) OR (body weight)) OR (weight management)) OR (Body Composition)) OR (Waist circumference) | 1,106,110 |
| #2 | Search: (((((adolescent) OR (child)) OR (teen)) OR (youth)) OR (minors)) OR (Pediatric) | 4,427,330 |
| #1 | Search: (((((((((((((((((((((((Wearable Electronic Devices) OR (Device, Wearable Electronic)) OR (Devices, Wearable Electronic)) OR (Electronic Device, Wearable)) OR (Electronic Devices, Wearable)) OR (Wearable Electronic Device)) OR (Wearable Technology)) OR (Technologies, Wearable)) OR (Technology, Wearable)) OR (Wearable Technologies)) OR (Wearable Devices)) OR (Device, Wearable)) OR (Devices, Wearable)) OR (Wearable Device)) OR (Electronic Skin)) OR (Skin, Electronic)) OR (wristbands)) OR (smartwatches)) OR (Fitbit)) OR (Sports bracelet)) OR (physical activity tracker)) OR (accelerometer)) OR (electronic bracelet)) OR (Pedometer) | 82,370 |
