# Supplementary figures and images for: The Effectiveness of Wearable Devices as Physical Activity Interventions for Preventing and Treating Obesity in Children and Adolescents: Systematic Review and Meta-analysis
Source: JMIR Mhealth Uhealth. 2022 Apr 8;10(4):e32435. doi: 10.2196/32435 (PMC9034426; doi:10.2196/32435)

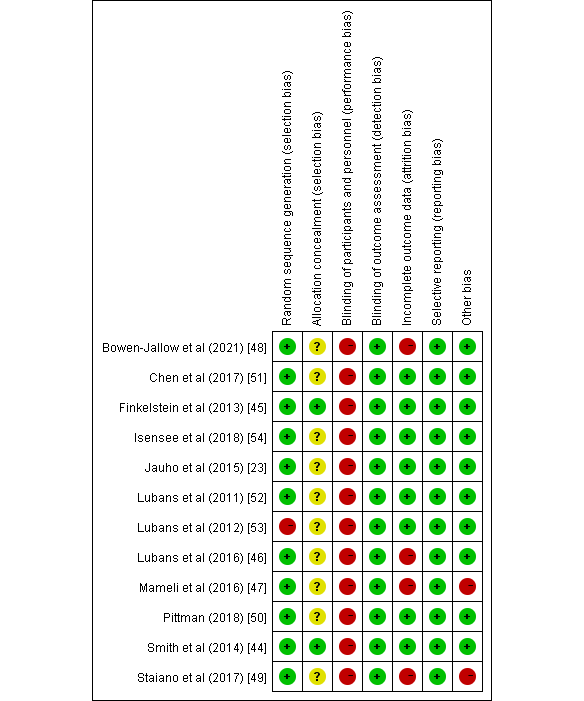

Supplement: Multimedia Appendix 3 [file mhealth_v10i4e32435_app3.png]
