# Supplementary material for: The Effectiveness of Wearable Devices as Physical Activity Interventions for Preventing and Treating Obesity in Children and Adolescents: Systematic Review and Meta-analysis
Source: JMIR Mhealth Uhealth. 2022 Apr 8;10(4):e32435. doi: 10.2196/32435 (PMC9034426; doi:10.2196/32435)
Supplement: Multimedia Appendix 4 [file mhealth_v10i4e32435_app4.docx]

Multimedia Appendix 4. Characteristics of Included Studies

| **Study** | **Participants** | **Duration** | **Wearable device** | **Intervention group** | **Control group** | **Related indicators** | **Result** |
| --- | --- | --- | --- | --- | --- | --- | --- |
| Lubans et al  2011 | Adolescent aged 14.3 years with normal-weight  (n=100) | 6-month | pedometers | The intervention group implements the physical activity leaders (PALs) program, which includes setting a step count goal | usual daily activities | Body Weight  BMI  Body fat %  Waist circumference  BMI z-score | The changes in body weight, BMI, BMI z-score, body fat between the control group and the intervention group was statistically significant. However, the changes of waist circumference were not statistically significant in the intervention group and the control group. |
| Lubans et al  2012 | Adolescent aged 12-14 years with normal-weight  (n=357) | 12-month | pedometers | The intervention group were provided pedometers and were encouraged to use these resources to monitor their physical activity participation. | received regular curriculum | BMI  Body fat %  BMI z-score | The changes in BMI, BMI z-score, body fat between the control group and the intervention group was not statistically significant. |
| Finkelstein et al  2013 | Children aged 6-12 years with normal-weight  (n=285) | 9-month | pedometers | The intervention group wears a pedometer, sets a step goal and gives cash rewards. | usual daily activities | BMI | The BMI changes of the intervention group before and after the intervention were statistically significant, but the BMI changes of the intervention group and the control group were not statistically significant. |
| Smith et al  2014 | Adolescent aged 12–14 years with normal-weight  (n=361) | 20-week | pedometers | The intervention group implements the ATLAS project, which includes setting a step count goal. | usual daily activities | BMI  Waist circumference  Body fat % | The changes of three index were not statistically significant in the intervention group and the control group. |
| Jauho et al  2015 | Adolescent aged 17.9 years with normal-weight  (n=276) | 3-month | wrist-worn watch | The intervention group were given a wrist-worn watch and gave feedback MVPA time, steps. | wears wrist -worn watch but not gave feedback | Body Weight  BMI  Body fat%  Waist circumference | The changes in weight, BMI, waist circumference between the control group and the intervention group was nor statistically significant. However, the changes of Body fat were statistically significant in the intervention group and the control group. |
| Mameli et al  2016 | Adolescent aged 10-17 years with obesity  (n=30) | 3-month | wristband | The intervention group wears a wristband and Use an app that records food consumption | only Received dietary instructions | Body Weight BMI z-score | The changes of two index were not statistically significant in the control. group and the intervention group. |
| Lubans et al  2016 | Adolescent aged 12–14 years with normal-weight  (n=361) | 18-month | pedometers | The intervention group implements the ATLAS project, which includes setting a step count goal. | continued their normal life | BMI  BMI z-score Waist circumference | The changes of three index were not statistically significant in the intervention group and the control group. |
| Staiano et al  2017 | Children and adolescent aged 8–17 years with overweight or obesity  (n=105) | 10-week | pedometers | The intervention group implements the ‘‘Our Lifestyles, Our Lives’’ weight management project, group 1 only Pedometer, group 2 Pedometer + step goals. | implements weight project but no pedometers | BMI  BMI -Z  Body Weight | The changes of three index were not statistically significant in the control group and the intervention group 1. The changes of three index were statistically significant in the control group and the intervention group 2. |
| Chen et al  2017 | Adolescent aged 13 -18 years with overweight or obesity  (n=40) | 6-month | Fitbit Flex + mobile app | The mobile phone-based intervention had three components: use of the Fitbit Flex, online educational program and text messages. | encourage healthy eating and regular exercise | BMI  BMI z-score | The changes of two index were statistically significant in the intervention group and the control group. |
| Isensee et al  2018 | Adolescent aged 12-16 years with normal-weight (n=1162) | 12-week | pedometers | Students in the intervention group wear a pedometer and are rewarded based on the number of steps completed. | continued their normal life | Body fat % | The changes of the body fat were the statistically significant in the intervention group and the control group. |
| Pittman  2018 | Adolescent aged 11-14 years with normal-weight  (n=102) | 10-week | wristband  activity trackers | The intervention group 1 wore activity tracker the group 2 wore the activity tracker and received text messaging. | continued their normal life and received educational text messaging | BMI  Body fat % | The changes of two index were no statistically significant in the control and the intervention group 1. The changes of BMI were statistically significant in the intervention group 2 and the control group. |
| Bowen-Jallow et al  2021 | Adolescent aged 12-18 years with overweight or obesity  (n=48) | 18-week | Fitbit | The intervention group wears a Fitbit, encouraged to walk at least 10,000 steps/day. | continued their normal life | BMI  Body Weight Waist circumference | The changes of three index were not statistically significant in the control group and the intervention group. |
